# AF1q is a universal marker of neuroblastoma that sustains N-Myc expression and drives tumorigenesis

**DOI:** 10.1038/s41388-024-02980-y

**Published:** 2024-02-27

**Authors:** Babak Oskouian, Joanna Y. Lee, Shahab Asgharzadeh, Ranjha Khan, Meng Zhang, Julia R. Weisbrod, Youn-Jeong Choi, Latika Puri, Ana E. Aguilar, Piming Zhao, Julie D. Saba

**Affiliations:** 1 Department of Pediatrics and the Helen Diller Family Comprehensive Cancer Center, University of California at San Francisco, San Francisco, CA USA; 2 Children’s Hospital of Los Angeles, University of Southern California, Los Angeles, CA USA; 3 Loma Linda University Children’s Hospital, Loma Linda, CA USA; 4 Arnold Palmer Hospital for Children, Orlando, FL USA

**Keywords:** Cell biology, Health sciences, Cancer

## Abstract

Neuroblastoma is the most common extracranial malignant tumor of childhood, accounting for 15% of all pediatric cancer deaths. Despite significant advances in our understanding of neuroblastoma biology, five-year survival rates for high-risk disease remain less than 50%, highlighting the importance of identifying novel therapeutic targets to combat the disease. *MYCN* amplification is the most frequent and predictive molecular aberration correlating with poor outcome in neuroblastoma. N-Myc is a short-lived protein primarily due to its rapid proteasomal degradation, a potentially exploitable vulnerability in neuroblastoma. AF1q is an oncoprotein with established roles in leukemia and solid tumor progression. It is normally expressed in brain and sympathetic neurons and has been postulated to play a part in neural differentiation. However, no role for AF1q in tumors of neural origin has been reported. In this study, we found AF1q to be a universal marker of neuroblastoma tumors. Silencing AF1q in neuroblastoma cells caused proteasomal degradation of N-Myc through Ras/ERK and AKT/GSK3β pathways, activated p53 and blocked cell cycle progression, culminating in cell death via the intrinsic apoptotic pathway. Moreover, silencing AF1q attenuated neuroblastoma tumorigenicity in vivo signifying AF1q’s importance in neuroblastoma oncogenesis. Our findings reveal AF1q to be a novel regulator of N-Myc and potential therapeutic target in neuroblastoma.

## Introduction

Neuroblastoma is the most common extracranial solid tumor of childhood, responsible for 15% of childhood cancer deaths [1]. It develops from neural crest, an embryonic tissue that gives rise to sensory, sympathetic, and parasympathetic neurons. Some neuroblastoma tumors spontaneously regress or differentiate, and low-risk neuroblastomas are associated with a 90–95% cure rate [2]. However, neuroblastoma can also present in an aggressive form that is relentless and resistant to multimodal therapy including high-dose myeloablative chemotherapy, radiation and stem-cell transplantation [3]. High-risk neuroblastomas are associated with a less than 50% five-year event-free survival rate, making identification of novel and more effective therapies a critical goal.

Myc family proteins are fundamental to the biology of neuroblastoma [4]. Amplification of *MYCN* is observed in ~25% of neuroblastomas and is associated with unfavorable outcome. Augmented *MYC* expression is responsible for aggressive behavior in other neuroblastomas [5]. Myc proteins are basic helix-loop-helix and leucine zipper transcription factors that act through transcriptional and target-gene independent mechanisms to control cell proliferation, apoptosis, metabolism and other cellular processes whose regulation undergirds normal homeostasis and whose deregulation is fundamental to carcinogenesis [6].

Under physiological conditions, Myc proteins are short-lived with a half-life of 15–30 min due to their rapid proteasomal degradation [7]. The AKT/GSK3β and Ras/ERK signaling pathways mediate two separate protein phosphorylation cascades that terminate in the Myc proteins themselves, ultimately controlling their ubiquitination on conserved lysine residues, thereby determining their fate [8]. To date, 18 ubiquitin ligases, 6 deubiquitinating enzymes and one SUMO protease have been shown to control ubiquitination and stability of Myc proteins [9]. In response to physiological growth signals, the half-lives of Myc proteins may be temporarily extended via interactions impinging upon AKT/GSK3β and/or Ras/ERK pathways or ubiquitin posttranslational modifications [8]. However, in neuroblastoma N-Myc or C-Myc protein expression levels are sustained through a variety of mechanisms. This pathway represents a potentially exploitable vulnerability in neuroblastoma.

*AF1Q* (ALL1-fused gene from chromosome 1q) also known as *MLLT11*, encodes a 90-amino-acid protein (AF1q) with no known enzymatic activity. AF1q was first identified in an infant with acute myelogenous leukemia (AML) harboring a t(1;11)(q21;q23) chromosomal translocation and was subsequently found to be overexpressed in hematologic malignancies as well as in several solid tumors [10–17]. In most of these malignancies, AF1q expression is a predictor of poor prognosis. In various cancer cell model systems, AF1q upregulation has been associated with enhanced proliferation, migration, and invasion [13, 14, 18–20]. Moreover, AF1q has been shown to activate the AKT pathway as well as being a component of the Wnt pathway by acting as a co-factor for TCF7 [14, 21]. AF1q is expressed primarily in neural crest-cell derived ganglia [22]. Ectopic AF1q expression can also promote neuronal differentiation in non-neuronal cells [23]. Despite its neurogenic properties and expression pattern, to our knowledge, a role for AF1q in neural tumors has not been reported.

In this study, we explored the potential role of AF1q in neuroblastoma. We discovered that AF1q is universally expressed in neuroblastoma tumors regardless of risk classification, as well as in *MYCN* amplified and *MYCN* non-amplified neuroblastoma cell lines. Silencing AF1q in neuroblastoma cells resulted in cell cycle arrest, apoptosis, and a profound reduction in tumorigenicity. Moreover, we demonstrate that AF1q protects N-Myc from ubiquitination and proteasomal degradation by impacting AKT/GSK3β and Ras/ERK signaling pathways. Our findings reveal AF1q to be a novel regulator of N-Myc and driver of neuroblastoma viability and tumorigenicity.

## Results

### AF1q is a universal marker of neuroblastoma

**Fig. 1:**
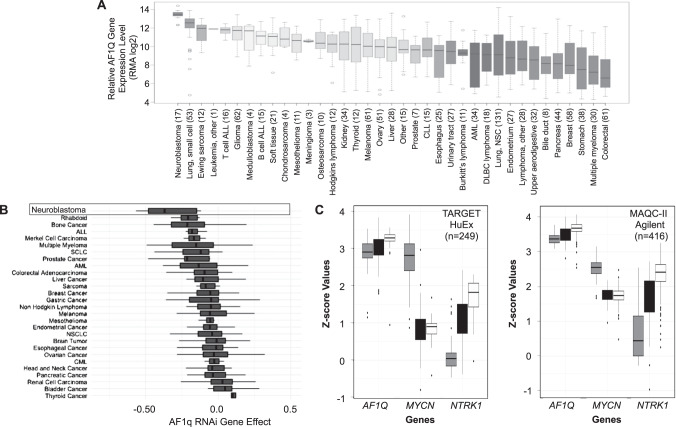
AF1q is a universal marker of neuroblastoma. **A** *AF1Q* expression in cancer cell lines represented in the Broad Institute Cancer Cell Line Expression (CCLE) Expression resource. Cell lines by cell/tissue of origin followed by number of cell lines per category in parenthesis. Chronic lymphocytic leukemia (CLL); Acute myelocytic leukemia (AML); Diffuse large B cell lymphoma (DLBC lymphoma); Lung, non-small cell (Lung, NSC). **B** Boxplot representations of data from DepMap project (DepMap_Public_21Q4 release) for DEMETER2 dependency score of cell lines from various cancer types. The DEMETER2 score is based on data from a cell depletion assay. A lower DEMETER2 score indicates a higher likelihood that the gene of interest is essential in a given cell line. A score of 0 indicates a gene is not essential; correspondingly -1 is comparable to the median of all pan-essential genes. Neuroblastoma cell lines (*n* = 9) have the lowest DEMETER2 score with enriched *p*-value < 7.4e-07 (significance obtained from DepMap_Public_21Q4). **C** Gene expression levels of *MYCN*, *NTRK1* and *AF1Q* in microarrays from two large datasets of human neuroblastoma tumor transcriptomes, represented as z-scores. Results show the relative gene expression in high-risk *MYCN* amplified (HRA, gray bars), high-risk *MYCN* non-amplified (HRN, black bars) and low-risk (LR, white bars) tumors. For TARGET HuEx dataset, total tumor number = 249; for HRA, HRN and LR, *n* = 68, 151 and 30, respectively; for MAQCII Agilent dataset, total tumor number = 416; for HRA, HRN and LR, *n* = 69, 71 and 281, respectively.

Based on its expression pattern, we suspected AF1q might also be expressed in human tumors of neural origin. Using the Broad Institute’s Cancer Cell Line Encyclopedia database [24], we compared relative *AF1Q* gene expression in cell lines derived from 37 different types of pediatric and adult malignancies (Fig. 1A). *AF1Q* was expressed at higher levels in neuroblastoma than all other tumor types examined. All seventeen neuroblastoma lines expressed *AF1Q* with low variability and at higher levels than all other tumor cell types. *AF1Q* was highly expressed in lines representing other neural tumors including medulloblastomas and gliomas. Similarly, analysis of *AF1Q* gene expression in cancer cell lines using the “Depmap” Cancer Dependency Map database (https://depmap.org/portal/) showed that *AF1Q* gene expression was higher in neuroblastoma than in any other cell line represented (Supplementary Fig. 1A). When *MYCN* amplified and non-amplified neuroblastomas were separated, both were still among the highest expressing cancer cell lines (Supplementary Fig. 2). Using immunoblotting, we confirmed strong AF1q expression in two *MYCN* amplified neuroblastoma lines (Kelly and Lan-5) (Supplementary Fig. 1B). AF1q protein expression was also readily detected in extracts of six neuroblastoma tumors, compared to undetectable expression in normal adrenal gland (Supplementary Fig. 1C). The Depmap’s main feature is the ability to systematically identify cancer vulnerabilities across cancer types by analyzing the impact of gene silencing and gene editing in an array of cancer cell lines. Analysis of cancer cell dependency on *AF1Q* revealed that neuroblastoma cells are more reliant upon *AF1Q* than any other cell line represented in the data set (Fig. 1B). This was true for both *MYCN* amplified and non-amplified tumors (Supplementary Fig. 3). These findings suggest the possibility that AF1Q represents an Achilles heel in neuroblastoma.

In silico analysis of the National Cancer Institute’s Therapeutically Applicable Research to Generate Effective Treatment (TARGET) program database (https://ocg.cancer.gov/programs/target) was performed to compare *AF1Q*, *MYCN* and *NTRK1* gene expression in 249 primary neuroblastoma tumors segregated into three clinical categories: (1) high-risk *MYCN* amplified; (2) high-risk *MYCN* non-amplified; (3) low-risk. *MYCN* amplification designates a high-risk status [25]. High *NTRK1* expression correlates with favorable clinical and pathological features and good outcomes [26]. Not surprisingly, *MYCN* expression was highest in tumors exhibiting *MYCN* amplification, compared to both high-risk and low-risk tumors lacking *MYCN* amplification (Fig. 1C and Supplementary Fig. 4A, B). Conversely, *NTRK1* expression was lowest in *MYCN* amplified tumors and highest in low-risk tumors. In contrast to the other two markers, which each designated a particular clinical risk group, *AF1Q* expression was consistently high in all neuroblastoma tumors. A similar pattern was observed when *AF1Q* gene expression was compared in 416 neuroblastoma tumors from the MicroArray Quality Control (MAQC)-II study of common practices for the development and validation of microarray-based predictive models (Fig. 1C) [27]. We conclude that AF1q is a universal marker of neuroblastoma tumors, likely explained by its presumed role as a neural differentiation gene.

### AF1q is required for neuroblastoma cell proliferation

**Fig. 2:**
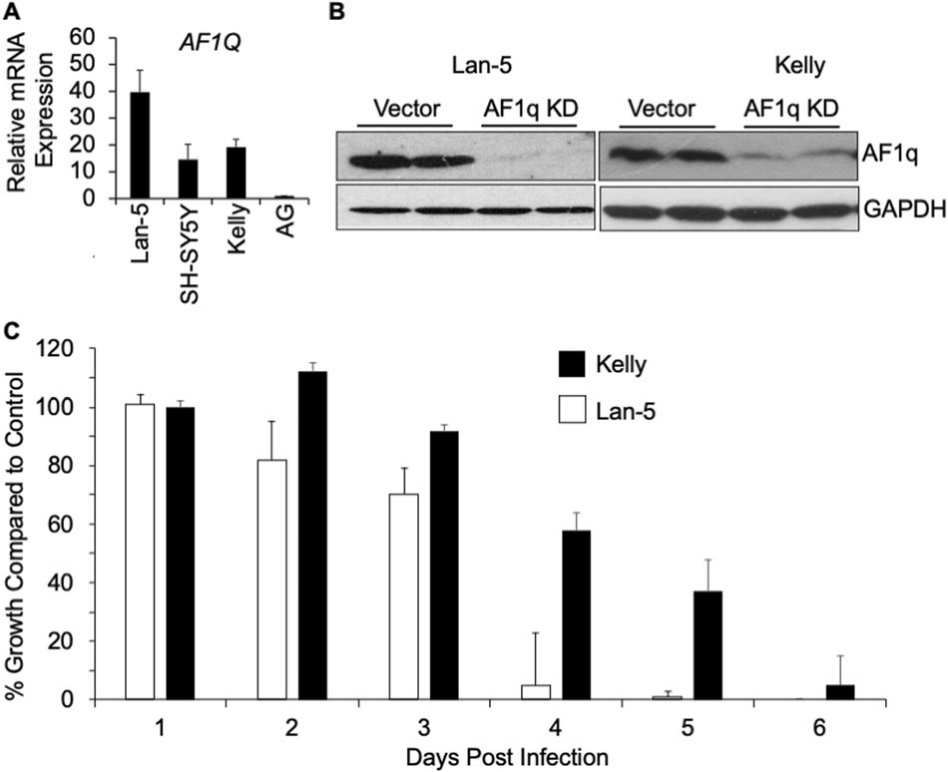
AF1q is required for neuroblastoma cell proliferation. **A** *AF1Q* gene expression in Lan-5, SH-SY5Y and Kelly neuroblastoma cells and adrenal gland (AG) control as measured by quantitative RT-PCR. **B** AF1q protein expression in paired Kelly and Lan-5 neuroblastoma cells with AF1q knocked down (AF1q KD) versus control. The extracts were made 72 h after transduction with shRNA lentiviruses. **C** Kelly (black bars) and Lan-5 (white bars) neuroblastoma cell proliferation determined on consecutive days after AF1q knockdown. For Lan-5 cells, day 1 vs. day 3 and beyond are significant (*p* < 0.05). For Kelly cells, day 1 vs. day 4 and beyond are significant (*p* < 0.05).

To confirm the dependency of neuroblastoma on AF1q, we set out to modulate its expression in neuroblastoma cell lines. First, we assessed *AF1Q* gene expression by qRT-PCR which confirmed our immunoblot data indicating high levels in Lan-5, Kelly (*MYCN* amplified) and SH-SY5Y (*MYCN* non-amplified) cell lines, whereas virtually no *AF1Q* expression was detected in normal adrenal gland (Fig. 2A). We silenced AF1q in Kelly and Lan-5 cells using a lentiviral system that expresses a small hairpin RNA (shRNA) specific to the 3′ untranslated region of the *AF1Q* transcript. Targeting AF1q in both Kelly and Lan-5 cells resulted in highly diminished AF1q protein expression compared to cells treated with shRNA control lentivirus (Fig. 2B). Compared to control cells, Kelly and Lan-5 cells lacking AF1q grew at reduced rates, detached from the culture plate, and could not be propagated beyond 5–7d, with Kelly cells taking a slightly longer time to reach complete cessation of growth compared to Lan-5 cells (Fig. 2C).

### In vivo tumorigenicity of neuroblastoma cells requires AF1q expression

**Fig. 3:**
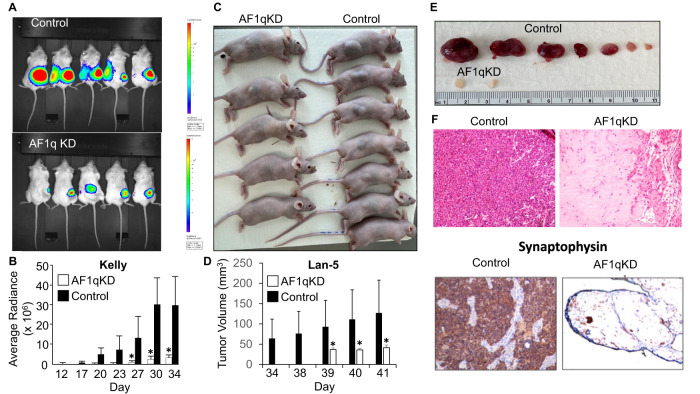
In vivo tumorigenicity of neuroblastoma cells requires AF1q expression. **A** Tumor bioluminescence of AF1q knockdown (AF1q KD) and control Kelly cell xenografts imaged at 30d after implantation into nude mice (*n* = 5/group). **B** Kelly cell xenograft bioluminescence quantification over time, showing average radiance with standard deviation. White bars represent AF1q KD; black bars represent control. *p < 0.05. AF1q knockdown compared to control. **C** Nu/Nu mice xenografted with Lan-5 cells were photographed 2 months after subcutaneous injection of control vs. AF1qKD cells (*n* = 5 AF1qKD; *n* = 7 control). **D** Lan-5 xenograft tumor size. *p < 0.05. **E** Xenograft tumors were extracted and photographed. **F** Lan-5 xenografts from vector control and AF1qKD cells stained with hematoxylin and eosin or synaptophysin immunohistochemistry.

Given its impact on cell growth in vitro, we asked whether AF1q may contribute to the tumorigenicity of neuroblastoma cells in vivo. Toward that end, we xenografted AF1q-depleted, and control, Kelly cells stably expressing a luciferase reporter, by subcutaneous injection into the flanks of immunodeficient mice. Tumor growth was then monitored by following bioluminescence signals using an IVIS Spectrum small animal imaging system. Control Kelly cell xenografts formed tumors that were detectible 20d after injection and grew rapidly until 34d, at which time the mice required euthanasia (Fig. 3A, B). In contrast, tumors derived from xenografted Kelly cells lacking AF1q were barely detectible at 27d after injection and exhibited 90 and 88% growth suppression compared to controls at 30d and 34d, respectively (Fig. 3A, B). Similarly, AF1q-KD Lan-5 cells injected into flanks of Nu/Nu mice failed to produce tumors after 2 months, whereas their control counterparts invariably produced tumors (Fig. 3C–E). The xenograft tumors arising from injected Lan-5 control cells were homogenous in morphology, exhibited high nuclear to cytoplasmic ratio, and were confirmed to be neuroblastomas by immunohistochemical staining for the neuroblastoma marker synaptophysin (Fig. 3F). In contrast, the small amount of tissue recovered at the injection site of mice injected with AF1q-silenced Lan-5 cells was mostly comprised of acellular material with sparse detectible cells that were also positive for synaptophysin and presumed to be neuroblastoma xenograft (Fig. 3F).

### Silencing AF1q in neuroblastoma cells induces apoptosis through the intrinsic pathway

**Fig. 4:**
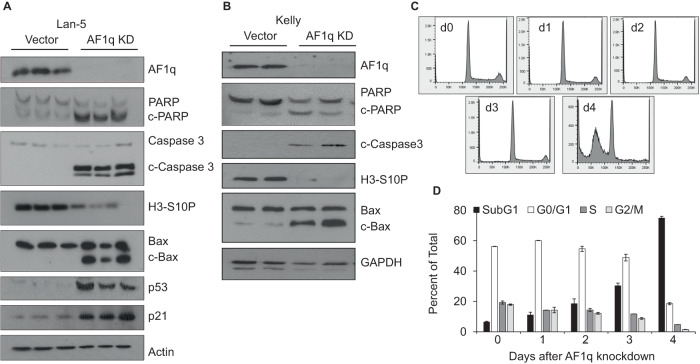
Silencing AF1q in neuroblastoma cells induces the intrinsic apoptotic pathway. **A** Immunoblot comparing PARP, caspase-3 and Bax cleavage products, histone 3 phosphorylated on serine 10 (H3- S10P), p53 and p21 in AF1q-silenced Lan-5 cells (AF1q KD) versus control (in triplicate). Actin is a loading control. **B** Immunoblot comparing the same markers in AF1q silenced and control Kelly cells (in duplicate) with GAPDH as the loading control. Lan-5 Cells were harvested 96 h, and Kelly cells 120 h post infection with shRNA carrying lentiviruses. **C** Cell cycle profiles of control cells and shAF1q treated cells at various times post infection (48–96 h) as indicated inside each frame are presented. **D** Quantitative representation of cell cycle analysis by percent total cells in each cell cycle phase.

To investigate whether AF1q depletion triggered programmed cell death, whole cell extracts of control and AF1q-depleted Lan-5 cells harvested 96 h after transduction were analyzed by immunoblotting to measure cleavage of the apoptosis markers poly (ADP-ribose) polymerase (PARP) and caspase-3. AF1q-silenced Lan-5 cells exhibited abundant PARP and caspase-3 proteolytic cleavage products, compared to low or undetectable levels in control cells (Fig. 4A). Apoptosis correlated with a block in cell cycle progression, as shown by reduced cellular levels of histone 3-serine10 phosphorylation (H3-S10P), a marker of mitosis [28]. Bax is a pro-apoptotic Bcl-2 family member that can induce mitochondrial pore transition and p53-dependent apoptosis [29]. The 18 kD Bax cleavage product was also elevated in AF1q-silenced Lan-5 cells (Fig. 4A). Similarly, when AF1q was silenced in Kelly cells, and cells harvested 120 h post transduction, proliferation was blocked, and apoptosis was induced (Fig. 4B). We conclude that silencing AF1q in neuroblastoma cells promotes cell death via the intrinsic apoptotic pathway. To corroborate apoptosis as the cause of cell death in AF1q-silenced neuroblastoma cells, cell cycle analysis was performed on Kelly cells at various times after AF1q knockdown and compared to cells treated with control shRNA. Cell cycle analysis revealed a marked decrease in the number of cells in all stages of cell cycle combined with a profound increase in the population of cells in the sub-G0 fraction as a function of the time post infection (Fig. 4C, D). These cumulative findings confirm that AF1q silencing induces mitochondrial apoptosis in neuroblastoma cells.

### Silencing AF1q in Lan-5 cells mediates apoptosis in a p53-dependent fashion

**Fig. 5:**
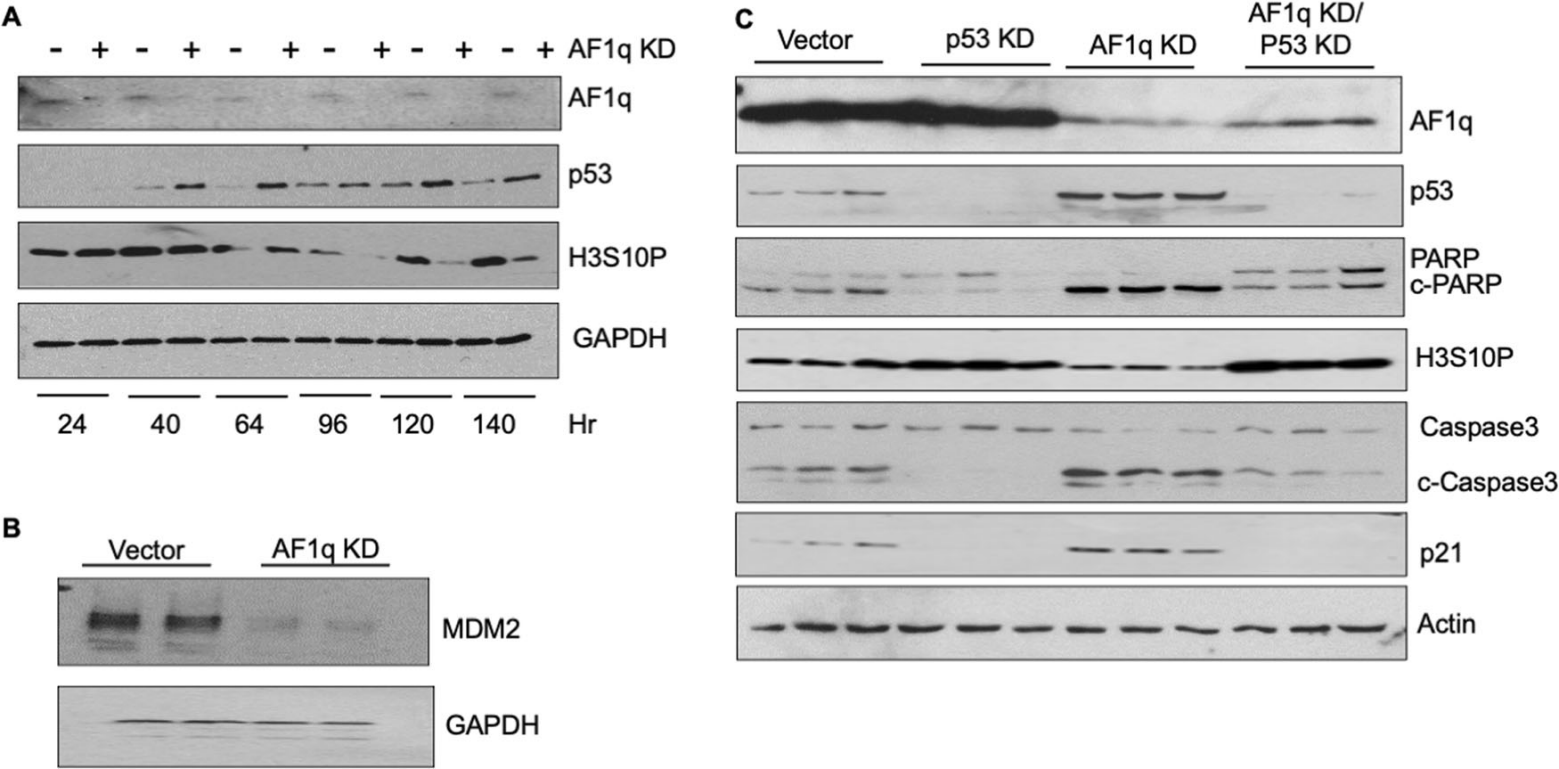
Silencing AF1q in Lan-5 cells mediates apoptosis in a p53-dependent fashion. **A** Time course demonstrating p53 induction and inhibition of cell cycle progression after AF1q silencing in Lan-5 cells. GAPDH is a loading control. **B** Silencing AF1q in Lan-5 cells results in downregulation of the p53 inhibitor, MDM2 (in duplicate). **C** Efficient silencing of p53 in Lan-5 cells maintains cell cycle progression (as shown by sustained H3-S10P expression) and prevents apoptosis (as shown by reduced cleavage of PARP and caspase-3) despite silencing of AF1q (in triplicate). All cells were analyzed 96 h after infection with shRNA carrying lentiviruses.

A time course study in which Lan-5 cells were analyzed following shAF1q-lentivirus infection indicated the reduction of AF1q, within 24 h of transduction (Fig. 5A). AF1q depletion was followed by upregulation of p53 at 40 h and cell cycle arrest at 96 h, as evidenced by loss of H3-S10P (Fig. 5A). Mouse double minute 2 homolog (MDM2) is a ubiquitin ligase which controls the cellular levels of p53 [30]. As depicted in Fig. 5B, MDM2 abundance was diminished in Lan-5 cells 96 h after AF1q silencing, which suggests that AF1q affects expression or stability of MDM2 and thereby affects p53 abundance in these cells.

To ascertain whether silencing AF1q mediates neuroblastoma cell death in a p53-dependent manner, we silenced p53 in (p53-wild type) Lan-5 cells concomitant with, or independent of, AF1q silencing, harvested cells 96 h after knockdown and performed immunoblotting on whole cell extracts. As shown in Fig. 5C, silencing p53 alone effectively eliminated both p53 and p21 expression in Lan-5 cells without altering AF1q expression. Silencing p53 also led to a slight increase in proliferation, as shown by an increase in H3-S10P levels. Consistent with our previous results, silencing AF1q led to upregulation of p53 and p21, a decrease in proliferation, and induction of apoptosis as shown by a marked increase in PARP and caspase-3 cleavage. In contrast, when AF1q and p53 were silenced in concert, proliferation proceeded and apoptosis was averted, as shown by levels of proteolytic cleavage products of PARP and caspase-3 that were indistinguishable from baseline levels. These cumulative findings suggest that AF1q silencing promotes apoptosis in a p53-dependent manner in Lan-5 cells by reducing MDM2 levels, thereby preventing proteasomal degradation of p53.

### Silencing AF1q in neuroblastoma cells reduces cellular N-Myc levels

**Fig. 6:**
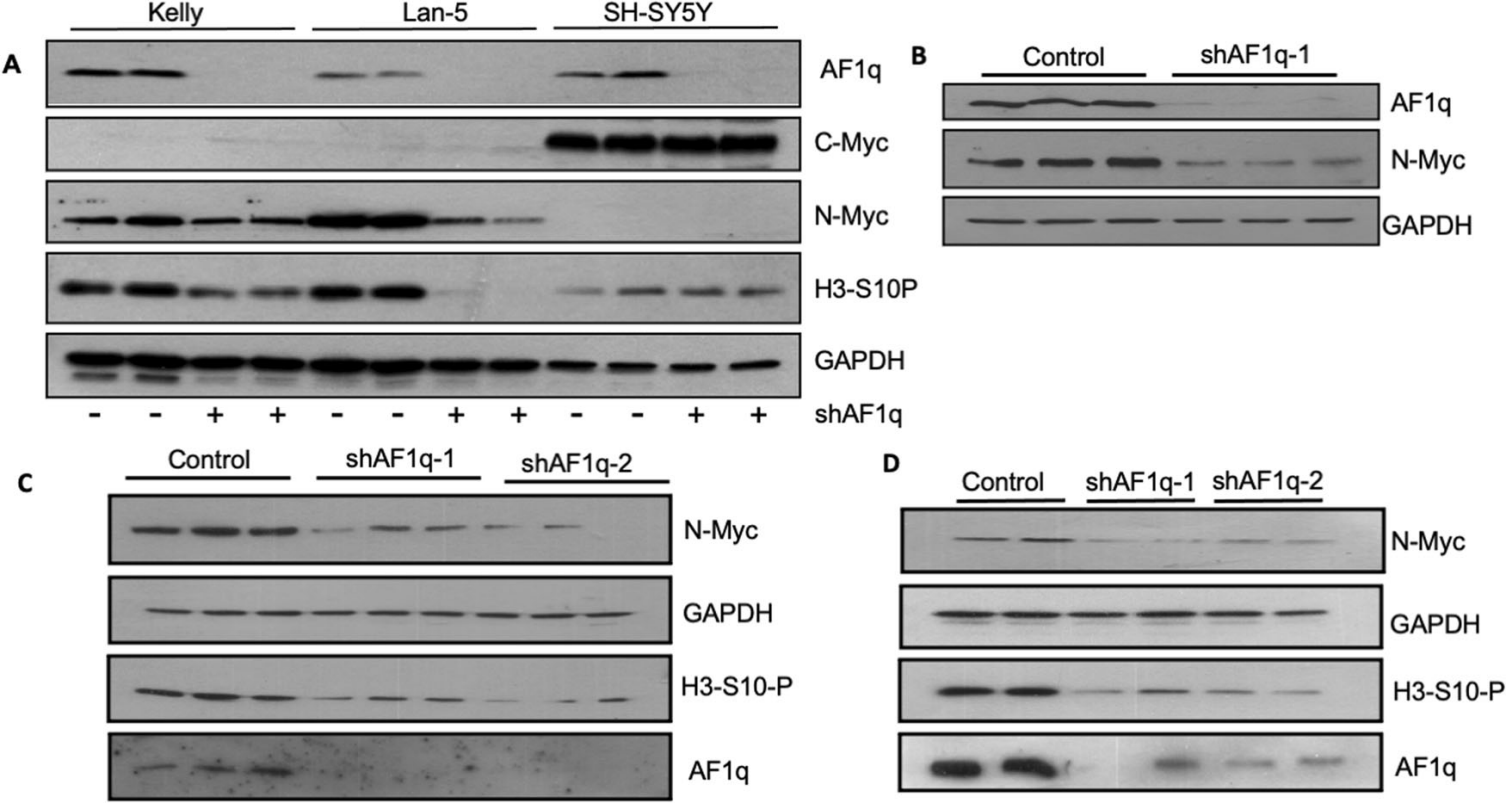
Silencing AF1q leads to downregulation of N-Myc in *MYCN* amplified neuroblastoma. **A** Immunoblot demonstrating the effects of AF1q silencing on N-Myc protein abundance in *MYCN* amplified Kelly and Lan-5 cells and C-Myc protein abundance in *MYCN* non-amplified SH-SY5Y neuroblastoma cells harvested 96 h after AF1q knockdown. N-Myc downregulation in Kelly and Lan-5 cells coincides with reduced proliferation as shown by H3-S10P levels (in duplicate). **B** N-Myc downregulation in Kelly cells harvested 120 h after AF1q knockdown (in triplicate). **C**, **D** Side by side comparison of the effectiveness of 2 different shRNAs targeting AF1q locus: shRNA-1 targets 3’UTR and shRNA-2 is against AF1Q coding sequence. Lan-5 cells (in triplicate, Part **C**) were harvested 96 h after infection. **D** Kelly cells (in duplicate) were harvested 120 h post transduction.

Because of the fundamental role played by N-Myc in neuroblastoma, we examined the impact of AF1q silencing on N-Myc in (*MYCN* amplified) Kelly and Lan-5 cells and on C-Myc in (*MYCN* non-amplified) SH-SY5Y cells. Silencing AF1q resulted in a profound reduction in N-Myc levels in Lan-5 cells harvested 96 h after knockdown (Fig. 6A). Similarly, Kelly cells showed reduced N-Myc levels at 96 h, with a more profound downregulation at 120 h (Fig. 6B). N-Myc downregulation coincided with cell cycle inhibition, as shown by reduced H3-S10P in both Lan-5 and Kelly cells (Fig. 6A). No changes in the abundance of C-Myc or H3-S10P were observed in SH-SY5Y cells under the same conditions (Fig. 6A). However, growth studies showed that, given enough time, even the SH-SY5Y cells exhibited a cessation of growth after AF1q silencing (Supplementary Fig. 5). The initial shRNA construct (shAF1q-1) used in these studies targets the 3’-UTR of AF1q. To rule out the possibility of off-target effects, a second shRNA lentiviral vector (shAF1q-2) which targets a sequence within the coding region of AF1q was constructed. Analyzing N-Myc expression in whole cell extracts of Lan-5 cells at 96 h (Fig. 6C) and Kelly cells at 120 h (Fig. 6D) post infection revealed that both constructs produced the same effects on N-Myc downregulation and His3-S10 phosphorylation.

Retinoic acid is a clinical agent used to treat neuroblastoma and is known to reduce N-Myc expression and promote neuroblastoma differentiation. The ability of AF1q silencing to inhibit neuroblastoma cell survival and reduce cellular N-Myc levels raised the possibility that retinoic acid might act in part by reducing AF1q expression. However, treating Kelly and Lan-5 cells with three forms of retinoic acid under conditions that reduced cellular N-Myc expression did not consistently affect AF1q levels, as shown in Supplementary Fig. 6.

### AF1q protects N-Myc by inhibiting its proteasomal degradation

The normally short half-life of N-Myc (~30 m) is due to its ubiquitination which targets it for degradation by the proteasome [31]. However, *MYCN* is also known to be regulated at the transcriptional level. To rule out an impact of AF1q silencing on *MYCN* gene expression, qRT-PCR was used to quantify *MYCN* mRNA levels in Kelly and Lan-5 cells after infection with AF1q shRNA and control virus (Supplementary Fig. 7A), at a time point when N-Myc protein levels were clearly reduced as shown by western blotting (Supplementary Fig. 7B). Under these conditions, we observed no significant change in *MYCN* expression, suggesting that the mechanism by which AF1q regulates N-Myc is post-transcriptional.

**Fig. 7:**
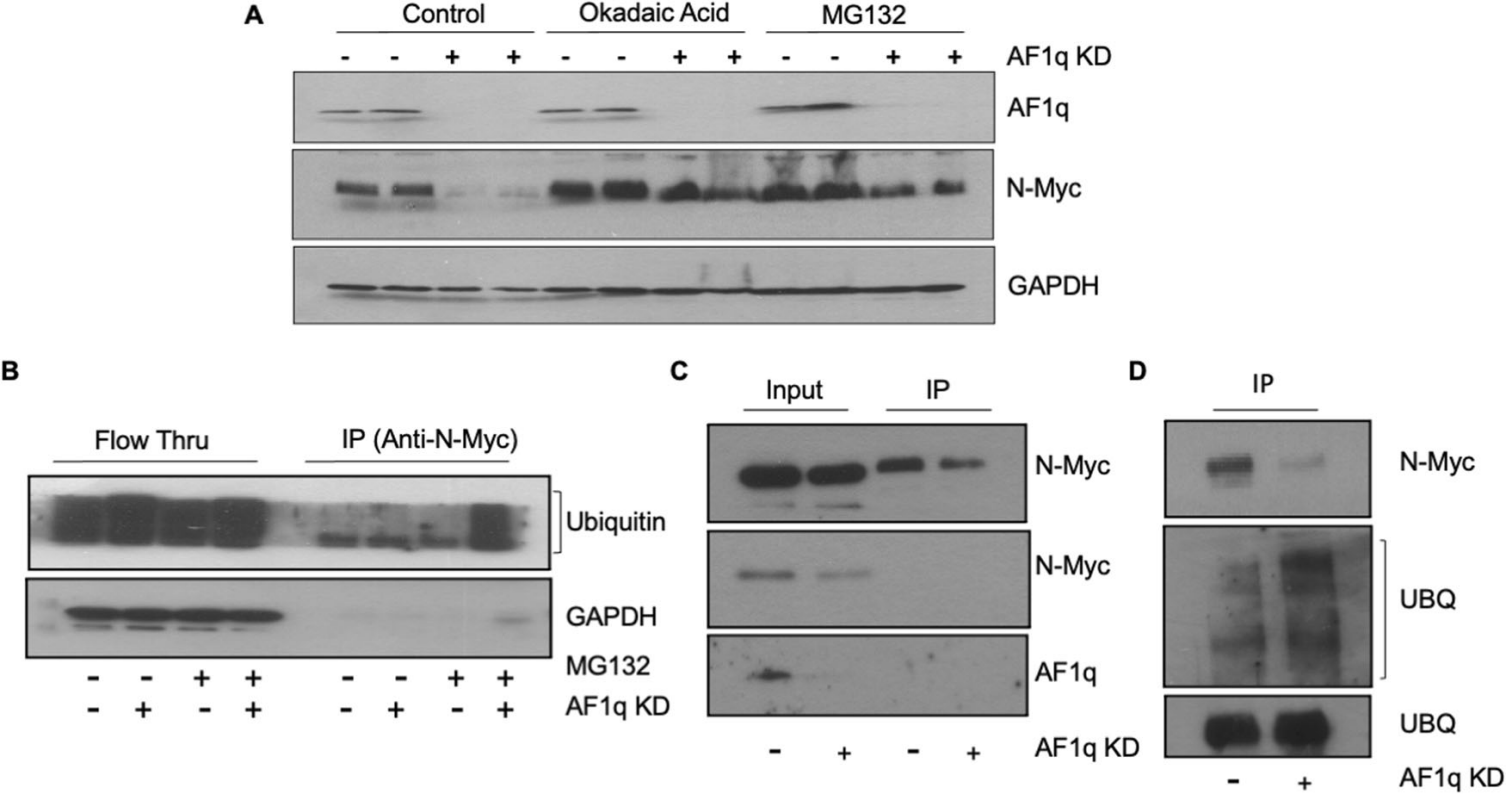
Silencing AF1q in neuroblastoma cells induces N-Myc proteasomal degradation. **A** Immunoblot shows N-Myc stabilization in Lan-5 cells pretreated with either 50 nM MG132 for 13 h prior to analysis or pretreated with 2 nM okadaic acid for 24 h prior to silencing AF1q (in duplicate). GAPDH is a loading control. Cells were harvested 72 h post shAF1q treatment. **B** Ubiquitinated forms of N-Myc increase in abundance in N-Myc pull-down precipitate from AF1q-depleted Kelly cells treated with MG132 (added 20 h before harvest) but not control Kelly cells treated with MG132. **C** AF1q knockdown reduces N-Myc in N-Myc pull-down precipitate from Kelly cell extracts. **D** Despite low amount of N-Myc protein in precipitate from AF1q knockdown Kelly cells, ubiquitinated forms of N-Myc are more abundant in the AF1q knockdown cells compared to control. Kelly cells were analyzed at 96 h post shAF1q treatment.

To investigate whether AF1q silencing causes N-Myc destruction by promoting proteasomal degradation in neuroblastoma, we treated Lan-5 cells with 50 nM of the proteasome inhibitor MG132. Without this treatment, AF1q silencing caused a nearly complete loss of N-Myc (Fig. 7A), whereas MG132 treatment abrogated the effect of AF1q silencing on N-Myc. Phosphorylation of Myc proteins on serine 62 has a stabilizing effect that prevents ubiquitination and degradation. Dephosphorylation of Myc proteins at serine 62 by protein phosphatase 2 A (PP2A) promotes Myc protein degradation [32]. To investigate the role of serine 62 phosphorylation in mediating AF1q’s impact on N-Myc stability, we inhibited PP2A using okadaic acid 24 h before shRNA treatment. Similar to the effect of proteasome inhibition, PP2A inhibition attenuated the ability of AF1q silencing to reduce N-Myc protein abundance (Fig. 7A). Together, these observations strongly suggested that AF1q protects N-Myc from proteasomal degradation. To further investigate this possibility, we sought to directly confirm the effect of modulating AF1q expression on N-Myc threonine 58 and serine 62 phosphorylation status. Toward that end, Lan-5 cells were treated with shRNA against AF1q for 24 h, at which time they were harvested and the phosphorylation status of N-Myc determined. As shown in Supplementary Fig. 8A, silencing AF1q in Lan-5 cells was associated with an increase in phosphorylation of N-Myc at threonine 58, a post-translational modification associated with N-Myc degradation. Further, co-transfection of HEK293T cells with constructs expressing N-Myc and either AF1q or a GFP control showed that AF1q overexpression resulted in an increase in the abundance of N-Myc phosphorylated at serine 62 (Supplementary Fig. 8B), a form of N-Myc that exhibits resistance to proteasomal degradation.

To directly interrogate the impact of AF1q on N-Myc ubiquitination, we compared the level of ubiquitinated forms of N-Myc in Kelly cells in which AF1q was silenced versus control cells in an immunoprecipitation experiment. Some aliquots were treated with MG132 to prevent immediate degradation of ubiquitinated N-Myc. In the absence of MG132 very little ubiquitinated N-Myc protein was detected in the pull-down material, even when AF1q was silenced (Fig. 7B). However, when MG132 was used to slow the proteasomal degradation of ubiquitinated proteins, a massive increase in ubiquitinated forms of N-Myc was detected in AF1q-silenced Kelly cell extracts compared to control, indicating that AF1q prevents N-Myc ubiquitination in neuroblastoma. To confirm these results while avoiding the complicating factor of proteasome inhibition, we repeated the pull-down experiment in Kelly cells harvested at an earlier time point after AF1q knockdown (in the absence of MG132). As expected, AF1q silencing reduced the amount of N-Myc protein that could be recovered in the pull-down precipitate (Fig. 7C, D). Remarkably, despite the low amount of N-Myc in AF1q-silenced Kelly cells, the amount of ubiquitinated forms of N-Myc in cells lacking AF1q far exceeded that of the control cells (Fig. 7D).

### AF1q protects N-Myc from degradation by modulating AKT/GSK3β and Ras/ERK signaling

**Fig. 8:**
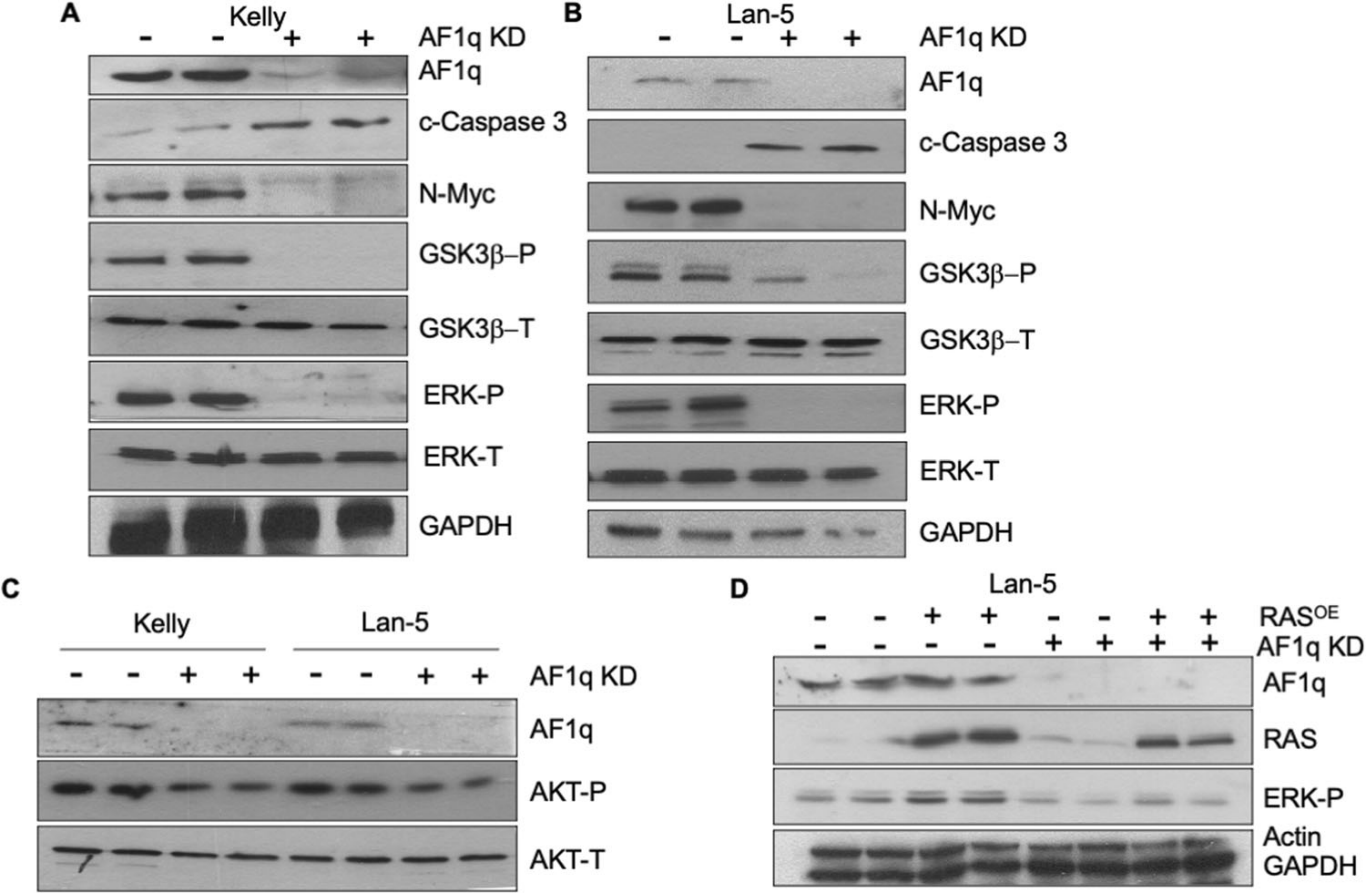
AF1q protects N-Myc from degradation by modulating AKT/GSK3β and Ras/ERK signaling. **A** Immunoblot showing reduced levels of N-Myc, phospho-ERK (ERKP, active form) and phospho-GSK3β (GSK3βP, inactive form) but no effect on total ERK (ERKT) or total GSK3β (GSK3βΤ) in Lan-5 cells depleted of AF1q compared to control. GAPDH is a loading control. **B** Similar results for AF1q silenced and control Kelly cells. **C** Immunoblot showing reduced levels of phospho-AKT (AKTP, active form) but no effect on total AKT (AKTT) in Lan-5 and Kelly cells treated with AF1q knockdown compared to corresponding controls. **D** Immunoblot showing expression of Ras, AF1q and phosphorylated ERK (ERKP, active form) in Lan-5 cells transduced with either oncogenic Ras alone or in combination with AF1q knockdown (AF1q KD). Actin and GAPDH are loading controls. Lan-5 cells were harvested 96 h and Kelly cells 120 h post transduction with shAF1q.

We next explored the mechanism by which AF1q protects N-Myc from ubiquitination. Ras signaling is known to promote N-Myc protein stability through downstream Ras/ERK and AKT/GSK3β signaling cascades. This bifurcated pathway ultimately controls the phosphorylation of N-Myc on threonine 58 (which promotes its ubiquitination) and serine 62 (which prevents its ubiquitination thus increasing its half-life) [8]. Whereas AKT and ERK activation stabilize N-Myc, GSK3β activation opposes AKT signaling and promotes N-Myc degradation [33]. In AF1q-silenced Kelly cells, we observed an almost complete loss of active (Thr202/Tyr204-phosphorylated) ERK as well as the inactive form of GSK3β (Ser9/21-phosphorylated) (Fig. 8A), whereas total ERK and GSK3β protein levels were unaffected by AF1q status. These findings coincided with a marked reduction in N-Myc levels and an increase in apoptosis, as shown by caspase-3 cleavage (Fig. 8A). Similar results were obtained in AF1q-silenced Lan-5 cells (Fig. 8B). Further, in both Kelly and Lan-5 cells, the active form of AKT (phosphorylated on Ser473) was diminished by AF1q silencing, whereas total AKT levels were unaffected under the same conditions (Fig. 8C). Finally, to interrogate the impact of AF1q silencing on Ras signaling, we infected Kelly cells with a lentivirus overexpressing oncogenic RasG12V (a constitutively active *HRAS* mutant), followed by superinfection with AF1q-targeted shRNA or control virus. As shown in Fig. 8D, Ras overexpression activated ERK in control cells. In contrast, overexpression of Ras in Kelly cells lacking AF1q failed to activate ERK. These findings demonstrate that AF1q stabilizes N-Myc by impacting Ras/ERK and AKT/GSK3β signaling cascades downstream of Ras (Supplementary Fig. 9). Further, we have shown that Ras is unable to activate ERK and protect N-Myc in the absence of AF1q expression.

## Discussion

The major significance of our study is the novel discovery of *AF1Q* as a universal and essential oncogene in neuroblastoma. Previous reports have demonstrated that AF1q is regulated by the neurogenic transcription factor REST, is expressed in the developing central and peripheral nervous systems, is a pan-neuronal marker in post-mitotic neurons and appears capable of directing neural differentiation in non-neural cell types [22, 23, 34]. Further, AF1q has well-established roles in carcinogenesis and prognostic significance in both leukemia and non-hematologic malignancies. Despite the findings that link AF1q to neurobiology and cancer, to our knowledge, a role for AF1q in tumors of neural origin had not been appreciated until now. Here we provide bioinformatics and experimental evidence for universally high levels of AF1q gene and protein expression in *MYCN* amplified and *MYCN* non-amplified neuroblastoma tumors and tumor-derived cell lines. In fact, *AF1Q* expression in neuroblastoma surpasses its expression level in all other cancer cell lines, as shown by our analysis of the Broad Institute’s Cancer Cell Line Encyclopedia.

The universal expression of AF1q in neuroblastoma prompted us to hypothesize a crucial role for AF1q in neuroblastoma biology. By silencing AF1q in two well-characterized *MYCN* amplified neuroblastoma lines (Kelly and Lan-5) using a highly efficient lentiviral knockdown system, we demonstrated that, without AF1q expression, neuroblastoma cells stop propagating in culture, undergo cell cycle arrest, followed by apoptosis and are not capable of forming tumors in vivo. Our finding that neuroblastoma cells require AF1q expression for survival and tumorigenesis is further supported by the highly significant dependency score of *AF1Q* in *MYCN* amplified and non-amplified neuroblastoma cells revealed by analysis of the Depmap database.

Neuroblastoma’s dependency on *AF1Q* could be due to its presumptive role in neural differentiation. However, two findings counteract that argument. The first is that high *AF1Q* expression in neuroblastoma tumors does not correlate with better outcome, as would have been expected in a gene that promotes differentiation. In fact, nearly all neuroblastoma tumors express high levels of *AF1Q*, while patient outcomes vary widely in neuroblastoma (Supplementary Fig. 10). The other is the remarkable observation that global, constitutive disruption of *AF1Q* in mouse tissues including the brain and spinal cord is well tolerated and does not cause noticeable phenotypes or appreciably disrupt central and peripheral nervous system development (our unpublished observations). This strongly suggests that the malignant neuroblastoma cells depend on *AF1Q* for functions that are independent of the maintenance of neural identity.

Investigation into the cellular events occurring after AF1q silencing revealed that the loss of viability and tumorigenicity associated with AF1q silencing could be attributed to the induction of the intrinsic apoptotic pathway involving cleavage of PARP, caspase-3 and Bax proteins.

In Lan-5 cells, these events were dependent on p53, as p53 and its target-gene p21 were induced when AF1q was silenced, whereas a shRNA targeting p53 could rescue the cells from the effects of AF1q depletion as shown by their continued proliferation and the lack of any evidence of apoptosis. Further analysis revealed that AF1q knockdown reduced the abundance of the p53 ubiquitin ligase, MDM2, providing a possible mechanism for the induction of p53. Mutations in p53 are infrequent in neuroblastoma tumors, and many studies report an intact p53 pathway in neuroblastoma cell lines, evidenced by nuclear p53 accumulation after DNA damage, its proper binding to DNA and transcriptional activation of p53 target genes [35]. While most neuroblastoma tumors initially respond to chemotherapy, they often relapse after treatment. Examination of these tumors has revealed that relapse is frequently associated with p53 loss/mutation [36].

Interestingly, when AF1q is silenced in Kelly cells (which lack a functional copy of p53), they also undergo mitochondrial apoptosis. This suggests that AF1q can engage other tumor suppressor pathways that operate in lieu of p53 in these cells. For example, Meng et al have shown that one of the candidate compensatory tumor suppressors in p53-mutant cells such as Kelly cells could be TP73—a member of the p53 family of transcription factors involved in cellular responses to stress [37]. TP73 maps to chromosome 1p36, a region that is frequently deleted in neuroblastoma (but which remains intact in Kelly cells). Another potential candidate to compensate for loss of p53 is the third member of the family, p63 [38]. Our findings suggest that targeting AF1q could potentially circumvent the chemo-resistance of p53-deficient neuroblastoma tumors.

The dependence of *MYCN* amplified neuroblastoma cells on AF1q expression prompted us to investigate whether AF1q exerts an influence on N-Myc, a key driver in the pathogenesis of neuroblastoma—especially *MYCN* amplified neuroblastoma. We observed a marked decrease in N-Myc expression after AF1q was silenced. The stability of Myc family proteins is regulated by a conserved pathway involving phosphorylation at key serine and threonine residues, which determines the ability of ubiquitin ligases to ubiquitinate and thereby target N-Myc to the proteasome for degradation. However, neuroblastoma cells with high N-Myc expression have overridden these pathways. It is well-established that phosphatase PP2A, by dephosphorylating Serine 62 of Myc proteins, primes them for ubiquitination and subsequent degradation, provided that T58 is simultaneously phosphorylated [39]. Using inhibitors of PP2A and proteasome function and by monitoring the ubiquitination status of N-Myc, we established that silencing AF1q promotes N-Myc ubiquitination and proteasomal degradation in *MYCN* amplified neuroblastoma. The lack of any effect of AF1q silencing on *MYCN* mRNA levels suggests that the effect on N-Myc ubiquitination is the primary mechanism.

It is intriguing that in Lan-5 cells we see a decrease of MDM2 upon AF1q depletion. There have been reports of MYCN-MDM2 interactions. For example, Slack et al. reported that N-Myc controls both mRNA and protein levels of MDM2 and that inhibition of N-Myc leads to MDM2 depletion, p53 activation and apoptosis [40]. Interestingly, there are also reports of MDM2 affecting *MYCN* expression by stabilization of *MYCN* mRNA and increasing its translation [41, 42]. Thus, there seems to be a reciprocal induction of *MDM2* and *MYCN* genes in neuronal cells [43]. Since we have established that the effect of AF1q on N-Myc is primarily via facilitating its proteasomal degradation, it is tempting to speculate that a lowering of N-Myc is responsible for the decrease in MDM2 levels, the subsequent p53 upregulation, and the ensuing apoptosis in these cells.

Additional molecular analysis revealed that AF1q modulates the two major signaling pathways downstream of Ras known to control N-Myc proteolysis, namely AKT/GSK3β and ERK. We observed that AKT loses its activating phosphorylation marks after AF1q depletion. This is reminiscent of the findings of Hu et al., who showed a similar effect of AF1q on AKT activity in colorectal cancer [14]. We propose that in the case of neuroblastoma, inhibition of AKT activity blocks its ability to phosphorylate (and thereby inhibit) its substrate, GSK3β. Active, unphosphorylated GSK3β is then free to phosphorylate N-Myc on Threonine 58, marking it for ubiquitination. We also demonstrated that overexpression of AF1q results in higher levels of N-Myc serine 62 phosphorylation, consistent with the proposed mechanism. Both the Ras/ERK and PI3K/AKT cascades are well-established pro-tumorigenic and growth promoting pathways with many downstream targets and key roles in carcinogenesis, independent of their role in Myc protein regulation [44, 45]. As Ras mutations are the initiating genetic events in many cancers, our data demonstrating the loss of ERK activation downstream of Ras suggest the intriguing possibility that targeting AF1q may provide a useful therapeutic strategy in oncogenic Ras-driven cancers [46].

In contrast to effects of AF1q silencing in *MYCN* amplified neuroblastoma, in SH-SY5Y cells which do not express N-Myc but exhibit high levels of C-Myc expression, silencing AF1q caused no appreciable change in C-Myc levels. This is an unexpected result, since the regulation of Myc proteins by phosphorylation, ubiquitination and proteasomal degradation is highly conserved among Myc family proteins [9]. In addition, we have observed diminished levels of C-Myc as a function of AF1q depletion in a number of non-neuroblastoma cells (Unpublished data). Our results could be explained if AF1q silencing exerted an effect on C-Myc stability with different kinetics than that of N-Myc. Alternatively, AF1q may exert its effect in an N-Myc specific manner, such as by interacting with protein partners specific to N-Myc.

In conclusion, we reveal AF1q to be a universal neuroblastoma marker that serves as a viability factor and regulator of N-Myc stability. Despite years of research, it has proven difficult to devise strategies to control N-Myc expression which has long been considered an “undruggable” target in neuroblastoma. One class of compounds that reduce N-Myc expression are the retinoids. We did not find that retinoic acids drastically influence AF1q expression, which suggests the possibility that targeting AF1q could be used in combination with retinoids. More recently, targeting the ubiquitination of Myc family proteins has emerged as a promising strategy, with numerous modulators in development, several reaching clinical trials, and two achieving Food and Drug Administration approval for use in cancer (8). In that context, our findings that AF1q acts as a hitherto unknown regulator of N-Myc proteasome-dependent degradation have identified a potential new target for development of N-Myc destabilizing strategies.

AF1q is a small protein with no known enzymatic activity. Thus, it is surprising that it can impinge upon so many different signaling pathways. We speculate that AF1q achieves this feat by playing the role of a co-activator or co-repressor via physical interaction with different components of signaling pathways, thereby altering their activity, as has been documented for AF1q’s role in the Wnt pathway, where it acts a co-factor for TCF7 [21]. Identifying potential interacting partners of AF1q will shed more light on how this small protein can have such profound influence in cancer and may provide a strategy to target its activity for therapeutic purposes [47].

## Materials and methods

### Data sources

We performed in silico analysis of publicly available databases from four different sources:

1. Broad Institute’s Cancer Cell Line Encyclopedia (https://sites.broadinstitute.org/ccle/), which provides gene-centric robust multi-RNA average (RMA)-normalized mRNA expression data [24].
2. DepMap portal (https://depmap.org/portal/), which provides analytical and visualization tools to explore gene expression and gene dependencies as described in (https://www.biorxiv.org/content/10.1101/720243v1).
3. National Cancer Institute’s Therapeutically Applicable Research to Generate Effective Treatment (TARGET) program which provides sequence data, microarray data and de-identified clinical information of cancer patients generated by Affymetrix Human Exon 1.0 ST Array (https://ocg.cancer.gov/programs/target).
4. MicroArray Quality Control (MAQC)-II study of common practices for the development and validation of microarray-based predictive models which was profiled using Agilent 44 K expression microarrays [27].

The expression percentiles were computed based on cumulative distribution function calculated for each sample’s gene profile.

### Reagents

All trans, 13-cis and 9-cis forms of retinoic acid are from Fisher Scientific (Rockford, IL). Cell cycle arresting reagents (mimosine, nocodazole, thymidine) were from Sigma-Aldrich (St. Louis, MO).

### Cell culture and transfections

Lan-5 and Kelly (*MYCN* amplified) neuroblastoma cell lines were purchased from UCSF Tissue Culture Facility (San Francisco, CA). SH-SY5Y (*MYCN* non-amplified) neuroblastoma and HEK293T cell lines were purchased from ATCC (Rockville, MD). Lan-5 and Kelly cells were grown in RPMI 1640 supplemented with non-essential amino acids. SH-SY5Y cells were grown in DMEM-H21. HEK293T cells were grown in DMEM. All culture medium were supplemented with 10% FBS, 100 U/ml penicillin and 100 μg/ml streptomycin.

### Specimens

Tumor specimens were obtained from six patients eleven months to four years of age diagnosed with neuroblastoma (of variable *MYCN* status) at UCSF Benioff Children’s Hospital Oakland (CHO). Specimens were provided by the CHO Tissue Bank as de-identified samples. Biopsies were taken at the time of diagnosis and represent primary samples from chemotherapy-naive patients. All samples were obtained with informed consent utilizing an approved Institutional Review Board protocol.

### Plasmids and silencing constructs

The AF1q coding region is located at nt 886 to 1158 in the mRNA sequence. Two small hairpin RNAs (shRNA) were generated (Integrated DNA Technologies, San Diego, CA) and cloned into Age I and Eco RI sites of the pLKO.1 plasmid (Addgene plasmid # 10878). The first shRNA is designed to target the sequence 5′- GCCAGAGTTGAGTTCTATGTA-3′ which lies between nucleotides 1283 to 1303 within the 3′ untranslated region of human AF1q. The second shRNA has the sequence of 5′-CCACCTACAAGGTCAAAGACA-3′ and targets a region within the coding sequence of *AF1Q* spanning nucleotides 107–127 (where A of the ATG start codon is designated as 1). Following DNA sequence verification, infectious virions were produced in HEK293T cells by transfecting these cells using polyethylenimine (PEI) transfection with a PEI to DNA ratio of 3:1. The transfection mix contains three plasmids: pLKO.1 with the shRNA construct, plasmid pMD2.G (Addgene plasmid # 12259; http://n2t.net/addgene:12259; RRID:Addgene_12259) which encodes VSV-G envelope, and plasmid psPAX2 (Addgene plasmid # 12260; http://n2t.net/addgene:12260; RRID:Addgene_12260) which encodes HIV Gag, Pol, Rev and Tat. The latter two were a gift from Didier Trono. Twenty-four hours post transfection, the media is refreshed and 48 h later, the supernatant containing the viruses is filtered through a 0.45 µM PVDF membrane and stored at 4 °C. The cells to be infected are treated with a final concentration of 8 µg/ml protamine sulfate. The plasmid expressing a luciferase reporter pGL3-Basic-IRES was a gift from Joshua Mendell (Addgene plasmid # 64784; http://n2t.net/addgene:64784; RRID:Addgene_64784). Lentivirus expressing a shRNA specific to the 3′ untranslated region of human p53 was a gift from Bob Weinberg (Addgene plasmid # 19119; http://n2t.net/addgene:19119; RRID:Addgene_19119). pLenti CMV RasG12V Neo (w108-1) was a gift from Eric Campeau (Addgene plasmid # 22259; http://n2t.net/addgene:22259; RRID:Addgene_22259) [48].

### Immunoblotting

Immunoblotting was performed as described [49]. Signals were visualized using the SuperSignal West-Pico kit (Fisher Scientific, Rockford, IL) and quantified by densitometry using ImageJ software (NIH). Caspase-3 (Cat #9579), Bax (Cat #2774), p53 (Cat #9282), MDM2 (Cat #86934), p21 (Cat #2924), poly (ADP-ribose) polymerase (PARP)(Cat #9542), C-Myc (Cat #9402), ubiquitin (Cat #3933), ERK (Cat #4695) and p-ERK (Cat #4370) antibodies are from Cell Signaling Technology (Danvers, MA). Antibodies against GAPDH (Cat #sc-47724) and N-Myc (Cat #sc-53993) are from Santa Cruz Biotechnology (Santa Cruz, CA). Antibodies against AF1q (Cat #M01 AT1064a) were purchased from Abgent (now Abcepta), and those against H3-S10-P (Cat #06-570) were from Upstate Biotechnology (now Millipore Sigma). Antibodies against β-actin (Cat #A2228) are from Sigma-Aldrich (St. Louis, MO). Antibodies for N-Myc phospho-threonine 58 (Cat #P00026) and phospho-serine 62 (Cat #P00026-2) were from Boster Bio (Pleasanton, CA).

### RNA extraction, cDNA synthesis and quantitative real-time PCR (qRT-PCR)

RNA was purified using TRIzol followed by RNase-free DNAse digestion (Qiagen) followed by a cleaning step using the RNAEasy kit (Qiagen). cDNAs were synthesized with the High-Capacity cDNA Reverse Transcription kit (Thermofisher Scientific). All qRT-PCR reactions were performed in an ABI 7900HT apparatus using SYBR-Green Power master mix (Thermofisher Scientific) with default cycling conditions; results were analyzed with ABI SDS 2.4 software. At least three technical and two biological replicates were done for all qRT-PCR experiments, and values were analyzed with QuickCalcs (GraphPad Software:http://graphpad.com/quickcalcs/Grubbs1.cfm) to detect outliers; dissociation curves were checked and products were run in agarose gels to confirm amplification resulted in only one product. Relative mRNA levels were calculated by the 2^(−∆∆Ct) method using beta actin or GAPDH as normalization controls. Primers: AF1Q-Fw: 5′-TCAGATACAGCCACCTACAA-3′; AF1q-Rv: 5′-CCTTAGAGCAAGTCCAGTTC-3′; MYCN Fw: 5′-CACAAGGCCCTCAGTACCTC-3′; MYCN-Rv: 5′-TTCTCCACAGTGACCACGTC-3′; GAPDH-Fw:5′-TCAAGGCTGAGAACGGGAAG-3′; GAPDH-Rv:5′-CGCCCCACTTGAT-TTTGGAG-3′; ACTB-Fw:5′-CATGTACGTTGCTAT-CCGGC-3′;ACTB-Rv:5′-CTCCTTA-ATGTCACGCACGAT-3′.

### Cell proliferation

Cell proliferation was measured by colorimetric detection of reduction of [3-(4,5-dimethylthiazol-2-yl)-5-(3-carboxymethoxyphenyl)-2-(4-sulfophenyl)-2H-tetrazolium, inner salt; MTS] using the CellTiter 96 Aqueous Non-Radioactive Cell Proliferation Assay from Promega according to the manufacturer’s instructions.

### Cell cycle analysis

Cells treated with AF1q silencing or control shRNAs were harvested and washed with cold PBS. Cells were fixed with 70% ethanol in PBS, washed with cold PBS, and then resuspended in PBS containing RNase A (100 μg/mL) and 40 μg/mL propidium iodide and incubated at 37 °C for 30 min. The DNA content of cells was obtained using a FACScalibur flow cytometer and analyzed using a FlowJo program. Synchronization of cells at various stages was first established using chemical reagents to determine each cell cycle phase. Following the establishment of cell cycle synchronization at various stages, we then determined the effect of AF1q on the cell cycle progression and apoptosis.

### Animals

For Kelly cells xenograft studies, 6-week-old NOD-CB17-Prkdcscid immunocompromised female mice (Sz/J) (Jackson Labs Stock No: 001303) were injected subcutaneously in both flanks with Kelly neuroblastoma cells harboring a luciferase reporter. Briefly, 2 × 10^6^ cells were suspended in PBS, mixed in a 1:1 ratio with Matrigel (BD Biosciences, USA) and injected subcutaneously and unilaterally into the mouse flank. Mice were injected with D-Luciferin (Caliper Part #: 122796) at 165 mg/kg 20 min prior to imaging.

Xenografting of Lan-5 cells was done by subcutaneous injection of 2.5 × 10^6^ cells into a single flank of Nu/Nu Balb/C athymic mice (Charles River) as described above. As Lan-5 cells did not harbor a luciferase gene, the tumor sizes were examined after sacrificing the mice two months post injection. The injected cells had been transduced with either a scrambled shRNA or a shRNA targeting AF1q two days prior to injection. All animals were included in the analysis, and no randomization was done, nor blinding of the operator. These studies were performed in accordance with an approved CHORI Institutional Animal Care and Use Committee protocol.

### Histology

Xenograft tumors were formalin-fixed, embedded in paraffin, and 4 µm tissue sections were stained with hematoxylin & eosin. Immunohistochemistry was performed using antibodies to synaptophysin (Clone SY38; Cat MAB5258 from Millipore Sigma) at 1:50 dilution.

### Statistical analysis

A paired two-tailed t-test (assuming unequal variance) was used to test the significance between two groups. A Bonferroni correction was applied when estimating significance over time between two groups in tumorigenesis measurements in vivo. A *p* value of ≤0.05 was considered significant.

### Graphics

Graphics were created with BioRender.com.

### Supplementary information

Supplemental Material

## Data Availability

All data generated or analyzed during this study are included in this published article and its supplementary information files.
