# Supplementary material for: AF1q is a universal marker of neuroblastoma that sustains N-Myc expression and drives tumorigenesis
Source: Oncogene. 2024 Feb 27;43(16):1203–13. doi: 10.1038/s41388-024-02980-y (PMC11014797; doi:10.1038/s41388-024-02980-y)
Supplement: Supplementary file 1 — Supplemental Material [file 41388_2024_2980_MOESM1_ESM.docx]

Oskouian et al., Supplemental Material

**Table of Contents**

**Supplementary Figure 1. *AF1Q* gene expression in neuroblastoma cell lines in comparison to other cancer cell lines.**

**Supplementary Figure 2. *AF1Q* gene expression in neuroblastoma cell lines with *MYCN* amplified and non-amplified neuroblastoma lines segregated.**

**Supplementary Figure 3. Sensitivity of cancer cell lines to RNAi against AF1q.**

**Supplementary Figure 4. *AF1Q* expression in different risk groups of TARGET and MACQ-II neuroblastoma cohorts.**

**Supplementary Figure 5. Silencing AF1q in SH-SY5Y cells leads to cell growth inhibition.**

**Supplementary Figure 6. Effects of retinoic acid on AF1q and N-Myc expression in neuroblastoma cells**.

**Supplementary Figure 7. Silencing AF1q in Kelly cells does not appreciably affect *MYCN* mRNA levels.**

**Supplementary Figure 8. AF1q effects on phosphorylation status of N-Myc.**

**Supplementary Figure 9. Schematic model of AF1q’s effects in neuroblastoma.**

**Supplementary Figure 10. *AF1Q* expression does not correlate with event free survival in neuroblastoma.**

**
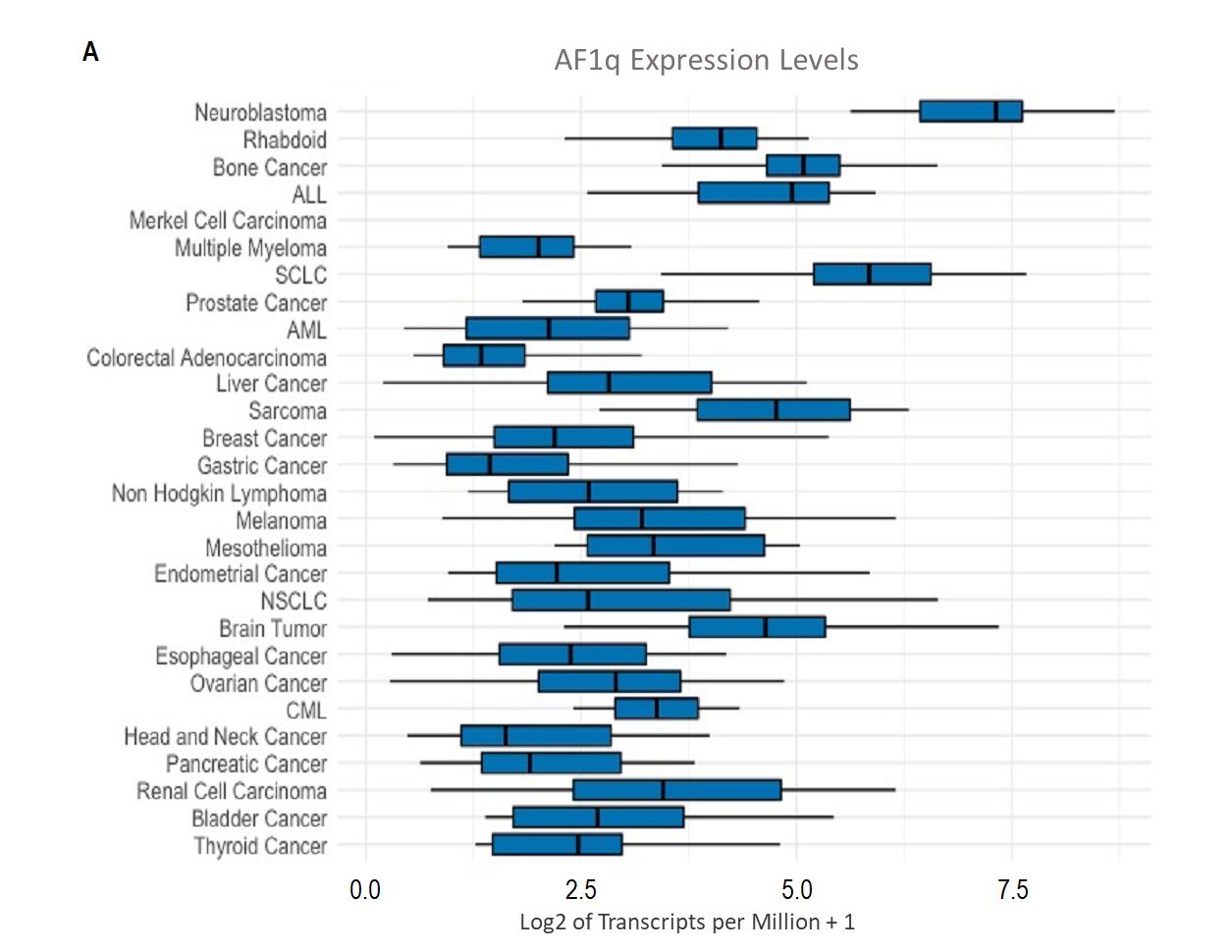
**

A

C

B

**Supplementary Figure 1. *AF1Q* gene expression in neuroblastoma cell lines in comparison to other cancer cell lines.** (A) Boxplot representations of *AF1Q* gene expression in different cancer cell lines; data extracted from the DepMap project database (DepMap_Public_21Q4 release). *AF1Q* expression is higher in the 9 neuroblastoma cell lines than all other cancer lines represented. Data are represented as the log2 [transcripts per million (TPM)+1] value. (B) AF1q protein expression in Kelly and Lan-5 neuroblastoma (*MYCN* amplified) cell lines with actin loading control. (C) AF1q expression in six neuroblastoma tumors (of variable MYCN status), with extracts of adrenal gland (AG) for comparison.

**
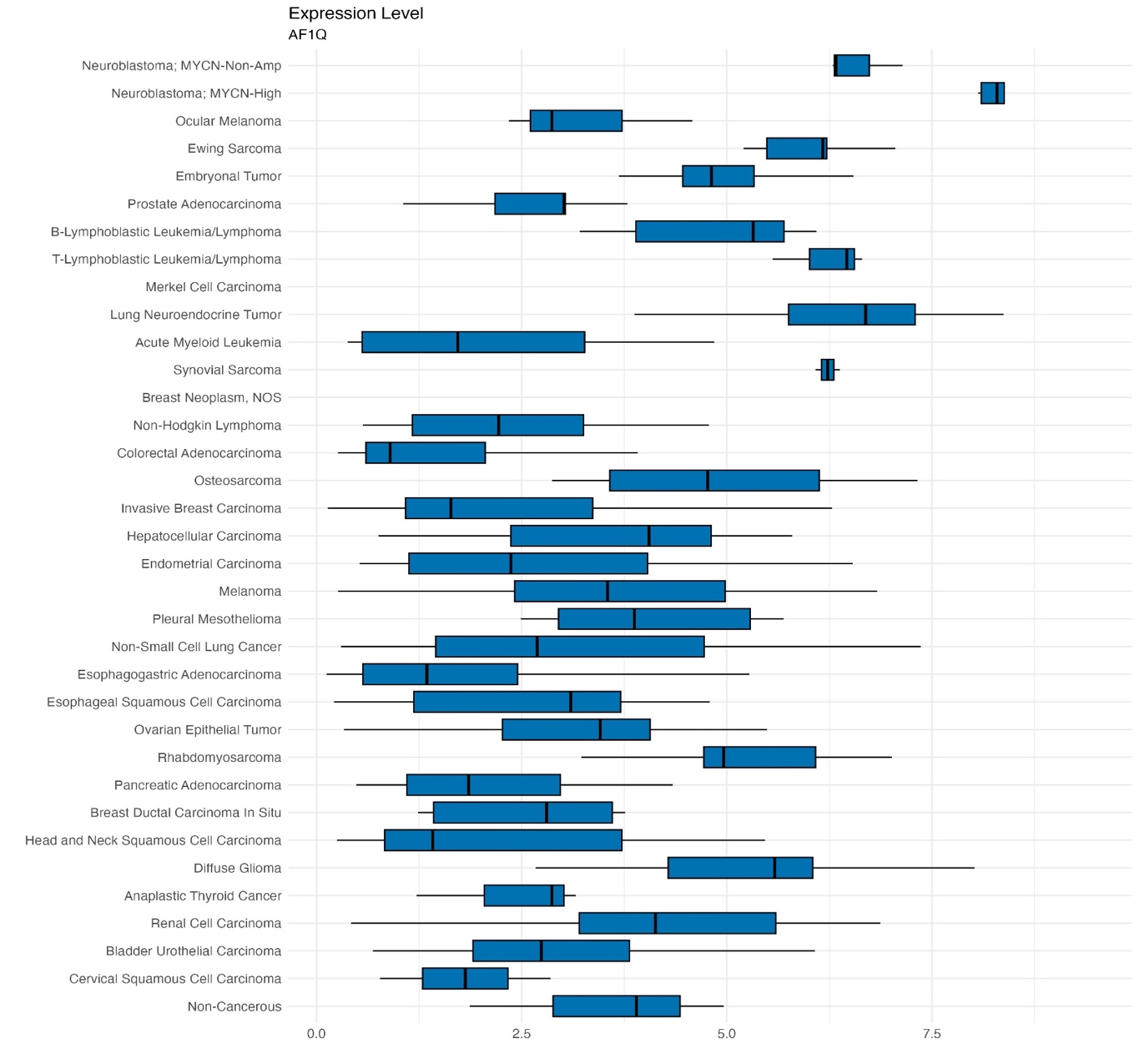
**

Log2 of Transcripts Per Million + 1

**Supplementary Figure 2. *AF1Q* gene expression in neuroblastoma cell lines with *MYCN* amplified and non-amplified neuroblastoma lines segregated.** (A) Boxplot representations of *AF1Q* gene expression as a function of *MYCN* amplification. Data extracted from the DepMap project database (DepMap_Public_22Q4 release).

**
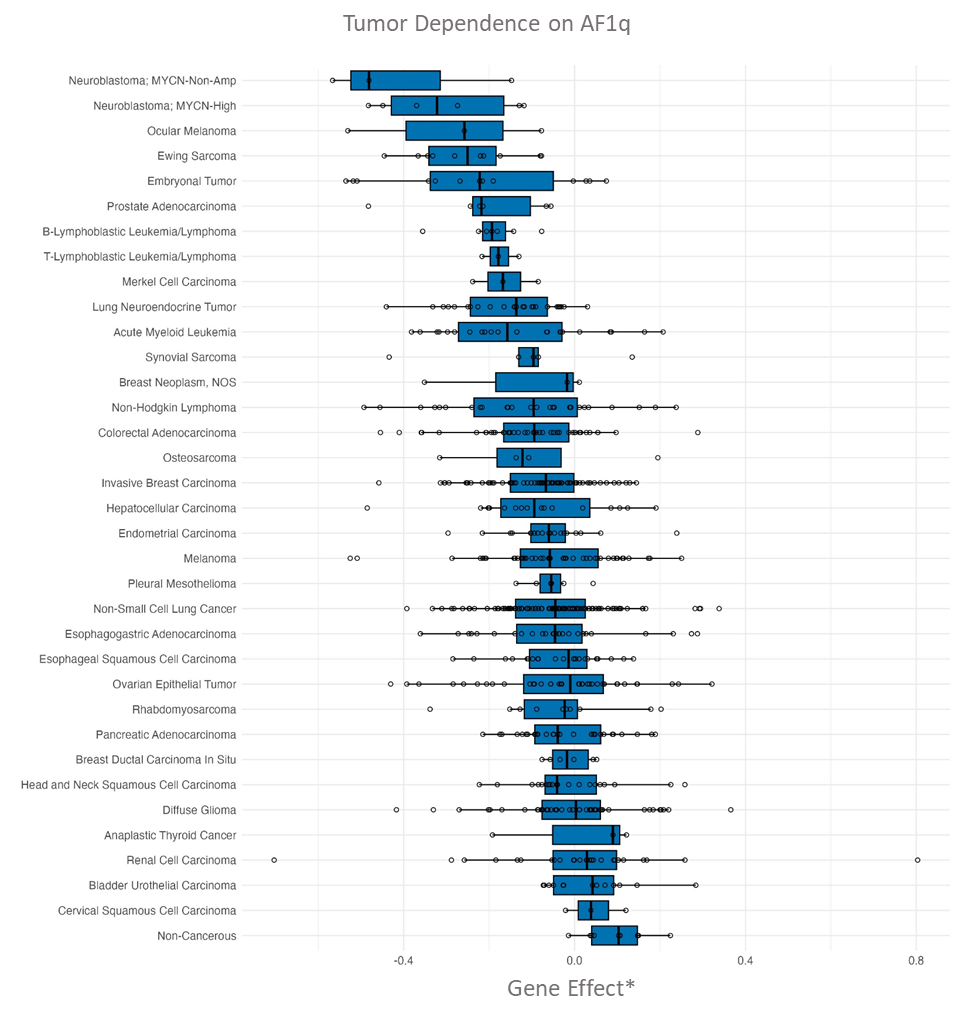
**

**Supplementary Figure 3. Sensitivity of cancer cell lines to RNAi against AF1q:** Box plot analysis of the effect of Knock down of AF1q on tumor cells and *MYCN* non-amplified vs. high *MYCN* neuroblastoma. DepMap project database (DepMap_Public_22Q4 release). *Gene Effect from Demeter 2 ranks dependence of cells on a particular gene. The lower the score, the higher the likelihood that a cell is dependent on that gene. A score of 0 represents a non-essential gene whereas a score of -1 is the median score for all common essential genes.

**Supplementary Figure 4. *AF1Q* expression in different risk groups of TARGET and**

**MACQ-II neuroblastoma cohorts.** Gene expression levels of *MYCN*, *NTRK1* and *AF1Q* in microarrays from two large datasets of human neuroblastoma tumor transcriptomes represented as: (A) gene expression values, and (B) percentile values. Percentile value is the value of empirical cumulative distribution function (ECDF), which returns the proportion of the original data points that are less than or equal to each of those values. This is useful to compare the values of a gene to other genes as ranked from 0 -100. Results show the relative gene expression in high-risk *MYCN* amplified (HRA, gray bars), high-risk *MYCN* non-amplified (HRN, black bars) and low-risk (LR, white bars) tumors. For TARGET HuEx dataset, total tumor number = 249; for HRA, HRN and LR, n = 68, 151 and 30, respectively; for MAQCII Agilent dataset, total tumor number = 416; for HRA, HRN and LR, n = 69, 71 and 281, respectively.

**Supplementary Figure 5. Silencing AF1q in SH-SY5Y cells leads to cell growth inhibition.** SH-SY5Y cells were treated with control or shRNA against AF1q for 24 hours after which the media was changed. The following day cells were trypsinized, counted and plated at a density of 50,000 cells per well. This was considered day 1. Every 2 days after that the cells from 3 wells per treatment were trypsinized and counted, and the average number of cells were plotted.


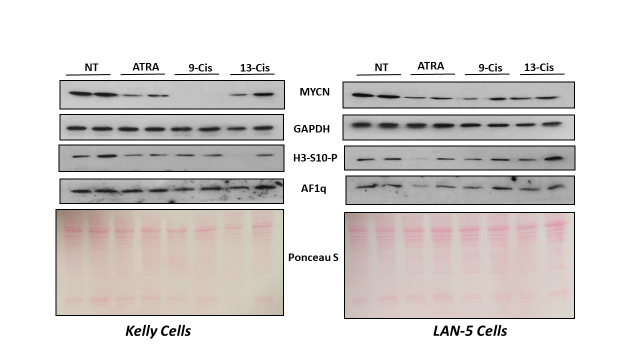


**N-Myc**

**Supplementary Figure 6. Effects of retinoic acid on AF1q and N-Myc expression in neuroblastoma cells.** Three forms of retinoic acid, namely all-trans retinoic acid (ATRA), 9-Cis–retinoic acid (9-Cis) and 13-cis-retinoic acid (13-Cis) were tested to assess their effects on AF1q expression. Lan-5 and Kelly-cells were treated with 10 µM of each of the drugs or were given no treatment (NT) for 48 hours, at which time the media was replaced with fresh media ± 10 µM retinoic acid. The cells were allowed to grow for another 48 hours and then harvested and analyzed. Ponceau staining for loading is shown below western blots.

A

**
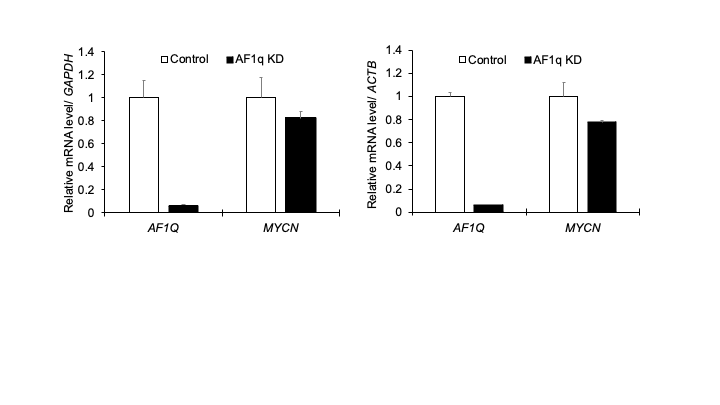
**

B

N-Myc

**
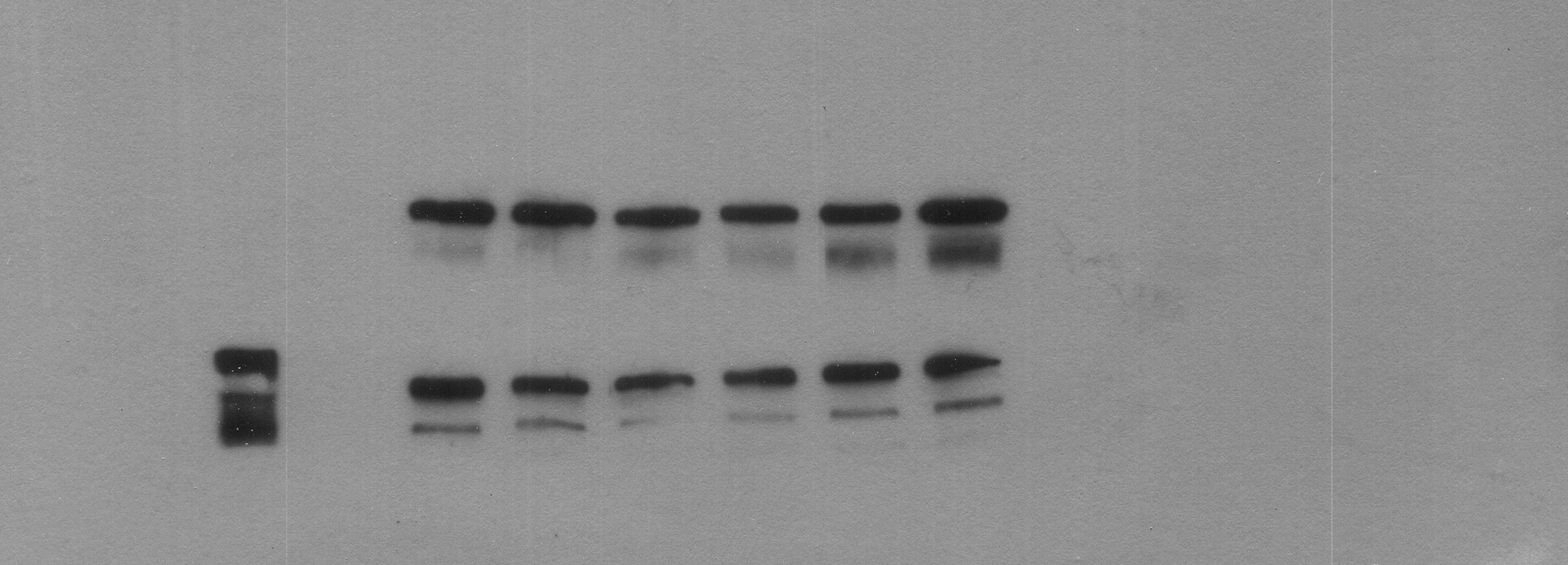
**

GAPDH

GAPDH

**
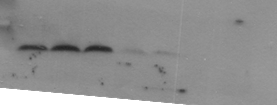
**

AF1q

**Supplementary Figure 7. Silencing AF1q in Kelly cells does not appreciably affect *MYCN* mRNA levels.** (A) Kelly cells were treated with either AF1q knockdown (AF1q KD) shRNA (black bars) or control lentivirus (white bars) and harvested 96h after infection. *AF1Q* and *MYCN* mRNA expression levels were measured by qRT-PCR as described in methods. *AF1Q* and *MYCN* levels were normalized to either *GAPDH* (left) or *ACTB* (right). Each experiment was performed in triplicate. For *AF1Q*, using either normalization method, control vs. AF1q KD, p < 0.05. For *MYCN*, using either normalization method, control vs. AF1q KD, no significant difference. (B) Western blotting demonstrates downregulation of AF1q and N-Myc protein levels at the same time point that *MYCN* mRNA was quantified.

**Supplementary Figure 8. AF1q effects on phosphorylation status of N-Myc protein.** (A) Lan-5 cells were treated with shRNA against AF1q for 24 hours, at which time they were harvested and the phosphorylation status of N-Myc at Thr58 was determined. (B) HEK293T cells were co-transfected with expression vectors for N-Myc plus either GFP (Control) or AF1q, harvested 60 hours after transfection, followed by N-Myc immunoprecipitation and analysis of N-Myc phosphorylation at Ser62 by Western blotting.


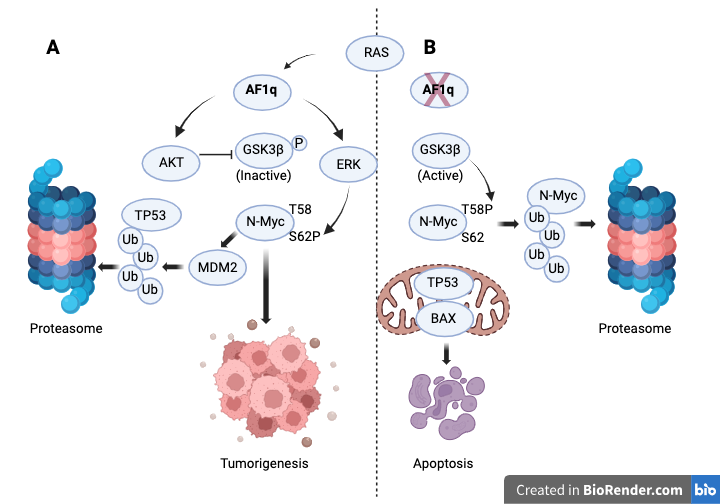


**Supplementary Figure 9. Schematic model of AF1q’s effect in neuroblastoma.** Two different patterns of cell signaling activity are present in neuroblastoma cells when AF1q is expressed or silenced. (A) In the presence of AF1q, AKT is active, and its substrate GSK3β is phosphorylated, rendering it inactive. ERK is active and phosphorylates N-Myc on serine 62, stabilizing it. MDM2 levels are high (possibly due to its known upregulation by N-Myc). MDM2 promotes the proteasomal degradation of p53. These conditions prevent apoptosis and promote tumorigenesis. In the absence of AF1q, AKT is inactive, leaving GSK3β unphosphorylated and active and therefore able to phosphorylate N-Myc on threonine 58. Further, in the absence of AF1q, Ras does not activate ERK, leaving N-Myc serine 62 unphosphorylated. These conditions render N-Myc susceptible to proteasomal degradation. MDM2 levels are low (possibly due to loss of N-Myc), and p53 is upregulated. Through an unknown mechanism, p53 is activated in the absence of AF1q and stimulates mitochondrial apoptosis, which is evident by BAX activation.

**Supplementary Figure 10. *AF1Q* expression does not correlate with event free survival in high-risk neuroblastoma.** Kaplan Meier event free survival curves showing lack of significant effect of *AF1Q* expression level on event free survival in (A) *MYCN* non amplified high-risk neuroblastoma and (B) *MYCN* amplified high-risk neuroblastoma. Results obtained from the TARGET neuroblastoma cohort.
